# Presence of the full-length *KIR2DS4* gene reduces the chance of rheumatoid arthritis patients to respond to methotrexate treatment

**DOI:** 10.1186/1471-2474-15-256

**Published:** 2014-07-28

**Authors:** Edyta Majorczyk, Andrzej Pawlik, Daria Gendosz, Piotr Kuśnierczyk

**Affiliations:** 1 Laboratory of Immunogenetics and Tissue Immunology, Ludwik Hirszfeld Institute of Immunology and Experimental Therapy, Polish Academy of Sciences, Weigla 12, 53-114 Wrocław, Poland; 2 Biochemistry and Physiology, Institute of Physiotherapy, Faculty of Physical Education and Physiotherapy, Opole University of Technology, Proszkowska 76, 45-758 Opole, Poland; 3 Pharmacokinetics and Therapeutic Drug Monitoring, Pomeranian University of Medicine, Powstańców Wlkp. 72, 70-111 Szczecin, Poland

**Keywords:** Rheumatoid arthritis, Treatment response, Methotrexate, KIR, KIR ligand

## Abstract

**Background:**

*KIR* genes coding for natural killer cell immunoglobulin-like receptors, KIR, influence the effector and regulatory function of NK cells as well as some subpopulations of T lymphocytes (e.g. CD4+CD28-KIR+) depending on presence of ligands (particularly HLA-C molecules). KIR-KIR ligand interaction may lead to the development of autoimmune disorders, including rheumatoid arthritis (RA). However, their role in the response of RA patients to methotrexate therapy is not known.

**Methods:**

*KIR* genes and KIR-ligand (*HLA-C C1/C2* allomorphs) genotyping was performed using the PCR-SSP method in 312 RA patients (179 classified as good responders and 133 as poor responders using DAS28 criteria). Thus, we evaluated the association of *KIR* genes and *HLA-C* allomorphs with the response to methotrexate (MTX) treatment.

**Results:**

We observed that patients possessing the full-length *KIR2DS4* (*KIR2DS4f*) gene had a lower chance of responding in comparison to *KIR2DS4f-*negative cases. This phenomenon was observed both in erosive disease (ED) and rheumatoid factor (RF) positive and in ED- and RF-negative patients. Interestingly, the observed effect of the *KIR2DS4f* gene was strongest in individuals possessing medium values (20-33 mm/h) of the erythrocyte sedimentation rate (ESR). Patients with high ESR values had low probability and, in contrast, patients with low ESR had a high probability of MTX response, and the presence of *KIR2DS4f* did not affect their outcome. Additionally, we show that the *KIR2DS4f* effect did not depend on the presence of either C1 or C2 allomorphs.

**Conclusion:**

Our results suggest that the response of RA patients with medium ESR values to MTX treatment may be dependent on the full-length *KIR2DS4* gene.

## Background

Rheumatoid arthritis (RA) is a chronic, relatively prevalent (about 1% of individuals in Caucasians) inflammatory disorder with a T cell-mediated autoimmune component [1]. Natural killer (NK) cells were also reported to contribute to RA [2]. These cells may act both directly, killing target cells, and indirectly, as either inducer or regulatory cells influencing innate and adaptive immunity (a “two-edged weapon”) [3].

The most polymorphic receptors of NK cells are encoded by killer cell immunoglobulin-like receptor (*KIR*) genes distributed differently in unrelated individuals. Since these receptors, depending on their structure, are either inhibitory or activating, their repertoire in a genotype affects activity of NK cells (and T cell subpopulations which also express KIRs, including T lymphocyte CD4 + CD28-KIR+) and, in consequence, susceptibility to different diseases including autoimmune disorders such as RA [4-6]. *KIR* haplotypes (i.e., sets of *KIR* genes on one chromosome) have been divided into group A, containing mostly inhibitory genes, and group B, containing several activating genes in addition to inhibitory ones. Moreover, the whole *KIR* region was divided into centromeric (A or B) and telomeric (A or B) halves, depending on the *KIR* gene content [7].

Ligands of KIRs, in most cases where known, are human leukocyte antigen (HLA) class I molecules which are extremely polymorphic. *KIR* and *HLA* genes are located on different chromosomes (number 19 and 6, respectively); therefore they are independently inherited and an individual may have KIRs with no ligands and vice versa. This affects the maturation of NK cells and the susceptibility of an individual to disease [8,9]. The most important ligands of KIRs are HLA-C molecules, which all fall into two groups: C1, with an asparagine residue in position 80, and C2, with a lysine residue in this position. KIR2DL1 and KIR2DS1 are receptors for the C2 epitope, whereas KIR2DL2 and KIR2DL3 bind C1 and also some C2 molecules. KIR2DS4 recognizes HLA-A*11 molecules as well as some C1 and C2. KIR2DL4 binds the “non-classical” class I molecule HLA-G. KIR3DL1 and possibly KIR3DS1 bind HLA-B with Bw4 epitope and those HLA-A alleles that possess the Bw4 motif. KIR3DL2 binds HLA-A*03,*11, and microbial CpG DNA [10]. In addition, both KIR3DL1 and KIR3DL2 bind HLA-B*27 homodimers [11,12].

As *KIR* and KIR ligand genotypes affect the immune response, and seem to influence the response to RA treatment using anti-TNF-α agents [13], we wondered whether these genotypes might also influence the outcome of MTX therapy of RA. MTX as a foliate antagonist is used in therapy of malignant disorders, but also suppresses the immune response in patients and in low doses was introduced for the treatment of RA because of its presumed anti-proliferative, immunosuppressive and anti-inflammatory properties [14]. However, MTX is not sufficient in all RA patients, and approximately 50-60% of RA patients could be classified as MTX responders [15-18]. It seems that the effectiveness of MTX treatment corresponds to the individual genetic background, particularly in genes encoding key molecules of methotrexate metabolism and toxicity [19,20]. However, clinically reliable markers of methotrexate efficiency are still sought.

## Methods

### Study subjects

Three hundred and twelve patients (253 females, 59 males; mean age, 58.0 ± 12.7 years, range 23-90; disease duration, 10.0 ± 8.8 years, range 1-50; age at onset 48.0 ± 13.1, range 10-80) were diagnosed with RA according to the criteria of the American College of Rheumatology (Table 1). Patients were recruited from the outpatient and inpatient population of the Department of Rheumatology, County Hospital in Szczecin, Poland. All subjects were Caucasian, from the Pomeranian region of Poland. The subjects enrolled in the study underwent routine biochemical blood analysis, and when required, assays for anti-cardiolipin antibodies, anti-nuclear antibodies, and immunological complexes. X-rays of the chest, hands, and feet were obtained in all patients and, when required, radiographs of other joints. These were interpreted by two expert radiologists. The evaluation of the subjects included physical examination, with particular focus on the pattern of joint involvement and the occurrence of extra-articular features (such as vasculitis, anemia, sicca syndrome, amyloidosis, organ involvement) and laboratory features, such as rheumatoid factor (RF) and anti-cyclic citrullinated peptide antibodies (anti-CCP) screened for using appropriate ELISA kits. Amyloidosis was diagnosed by histomorphology (skin and bowel or duodenum biopsy), vasculitis by histomorphology (skin biopsy) and angiogram.

**Table 1:** Characteristics of patients with rheumatoid arthritis

**Groups**	**Number of cases**	**Age**	**Disease duration**	**Age at onset**
	**(females/males)**	**(mean ± SD, range)**	**(mean ± SD, range)**	**(mean ± SD, range)**
All cases	312 (253/59)	58.0 ± 12.7, 23-90	10.0 ± 8.8, 1-50	48.0 ± 13.1, 10-80
Good responders	179 (143/36)	59.4 ± 12.4, 23-84	9.0. ± 7.3, 1-37	50.4 ± 12.3, 19-77
Poor responders	133 (110/23)	56.1 ± 12.9, 24-90	11.3 ± 10.0, 1-50	44.9 ± 13.5, 10-80

All patients included in this analysis had a standard methotrexate treatment with a regimen of oral 7.5 mg weekly, and with the dosage increasing to 20 mg weekly after 4 weeks, in combination with folic acid (1 mg daily). No patients received any other disease-modifying antirheumatic drug (DMARD), and no researchers’ involvement in the patient care was practiced. A good MTX response was noted for patients who were receiving MTX and had a disease activity score based on 28 joint counts (DAS28) of ≤2.5 at 6 months. Poor responders were defined as patients who were also receiving MTX but had a DAS28 of >2.5 [21,22].

The study was approved by the Committee of Ethical Affairs of the Pomeranian Medical University in Szczecin and written informed consent was obtained from all subjects.

### DNA isolation, KIR and KIR ligand typing

These procedures were performed as described previously [23,24].

### Statistical analysis

To investigate relationship between genetic, clinical and anthropological variables and probability of a good response to MTX treatment, a generalized linear model with binomial errors was used. The Akaike information criterion was used as a measure of fit of models.

A bootstrap approach was employed to estimate model coefficients and 95% confidence intervals. Odds ratio (OR) was computed as a measure of effect size. Arithmetic mean and standard deviation were calculated for continuous variables. Haplotype frequencies for KIR repertoire were estimated with maximum likelihood function [25]. The likelihood ratio statistic, LRS, was used to test the differences in haplotype frequencies between good responders and poor responders. *LRS*_*df* = *21*_ = 2(*LL*_good_ + *LL*_poor_ ‒ *LL*_combined_) and LRS is approximately a *χ*^2^_*df* = 21_. Results were regarded as statistically significant at p < 0.05.

## Results

A clinical response to MTX therapy was obtained for 57.3% of RA patients. When the frequencies of the *KIR* genes were compared between good responders and poor responders of RA patient groups, we observed an effect of only one *KIR* gene, full-length *KIR2DS4* (*KIR2DS4f*), on the response to MTX therapy. Namely, patients possessing this gene had a lower chance of responding (p = 0.0334, OR = 0.4344, 95% CI = 0.215, 0.987; Table 2). Additionally, patients with erosive disease (ED) and rheumatoid factor (RF) were less likely to respond to MTX, and those positive for *KIR2DS4f* had an even lower chance than those who were negative for *KIR2DS4f* but positive for both ED and RF (36.2% and 43.6% of good responders, respectively; Table 3). Similarly, RA patients negative for ED and RF also had a lower chance of responding to MTX therapy if they possessed *KIR2DS4f* (75% responding in comparison to 88% within *KIR2DS4f*-negative patients; Table 3). On the other hand, the presence of *KIR2DS4f* did not affect the formation of RF (p = 0.89), ED (p = 0.78), or other clinical parameters (data not shown).

**Table 2:** ***KIR *** **gene distribution and its effect on MTX response**

**MTX response**	***KIR*** **genes**										
	***2DS2***	***2DL3***	***2DL2***	***2DS3***	***2DL1***	***3DL1***	***3DS1***	***2DS5***	***2DS1***	2DS4f*	***2DS4d***
Good responders (N = 179)											
Presence (N)	107	155	103	68	178	171	59	52	71	55	156
Absence (N)	7	24	76	111	21	8	120	127	108	124	23
Frequency [%]	59.78	86.59	57.54	37.99	99.44	95.53	32.96	29.05	39.66	30.73	87.15
Poor responders (N = 133)											
Presence (N)	75	121	71	33	129	121	46	41	55	50	110
Absence (N)	5	12	62	100	84	12	87	92	78	83	23
Frequency [%]	56.39	90.98	53.38	24.81	96.99	90.98	34.59	30.83	41.35	37.59	82.71

*Good responders vs poor responders: p = 0.0334; OR = 0.43, 95%CI = 0.215-0.987.

**Table 3:** **Full-length *KIR2DS4 *** **interaction in MTX response dependent on bone erosion and rheumatoid factor presence**

**MTX responsiveness**		**Frequency (good responders/all)**	
**ED**	**RF**	***2DS4f*** **presence**	***2DS4f*** **absence**
+	+	36.2 (21/58)	43.6 (48/110)
+	-	70.0 (14/20)	76.7 (33/43)
-	+	72.7 (8/11)	72.4 (21/29)
-	-	75.0 (12/16)	88.0 (22/25)

+, clinical factor detected; -, clinical factor absent.

**Figure 1:**
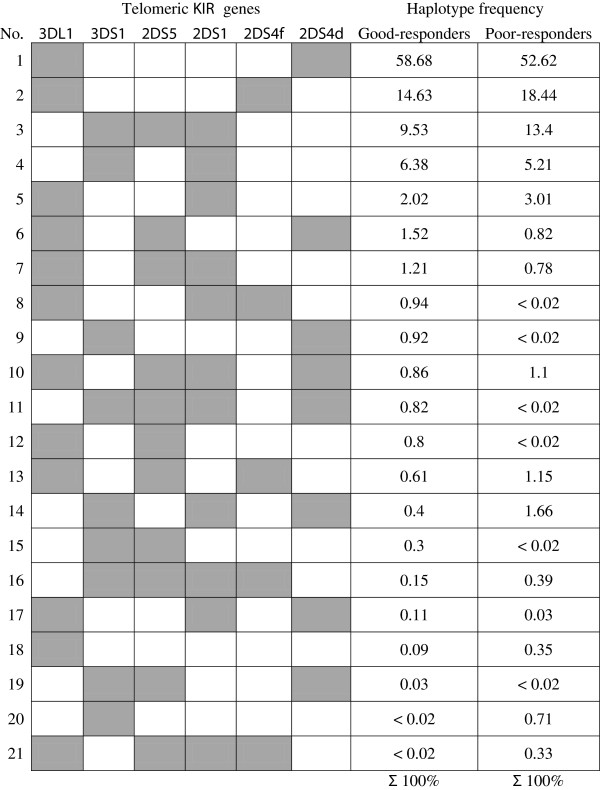
**Telomeric *KIR *haplotype profiles in MTX good responder and poor responder RA patient groups.** Grey box: gene presence; white box: gene absence. Estimation of *KIR* gene frequency based on haplotype distributions shown for *KIR3DL1* – 81.5% and 78.7%, for *KIR3DS1* 18.6% and 21.5%, for *KIR2DS5* 15.9% and 18.1%, for *KIR2DS1* 22.4% and 26.0%, for *KIR2DS4f* 16.4% and 20.3%, for *KIR2DS4d* 63.3% and 56.3%, in good responder and poor responder groups, respectively.

The *KIR2DS4f* gene is located in the telomeric half of the *KIR* locus [7]. However, we did not observe any differences between good and poor MTX responders in the estimated haplotype frequencies for the telomeric KIR repertoire (p = 0.29; Figure 1) despite the association of the *KIR2DS4f* gene with a poor response to MTX described above. Also, there were no differences in frequencies of centromeric (p = 0.71) or whole *KIR* locus (p = 0.96) genotypes between good and poor responders.

**Figure 2:**
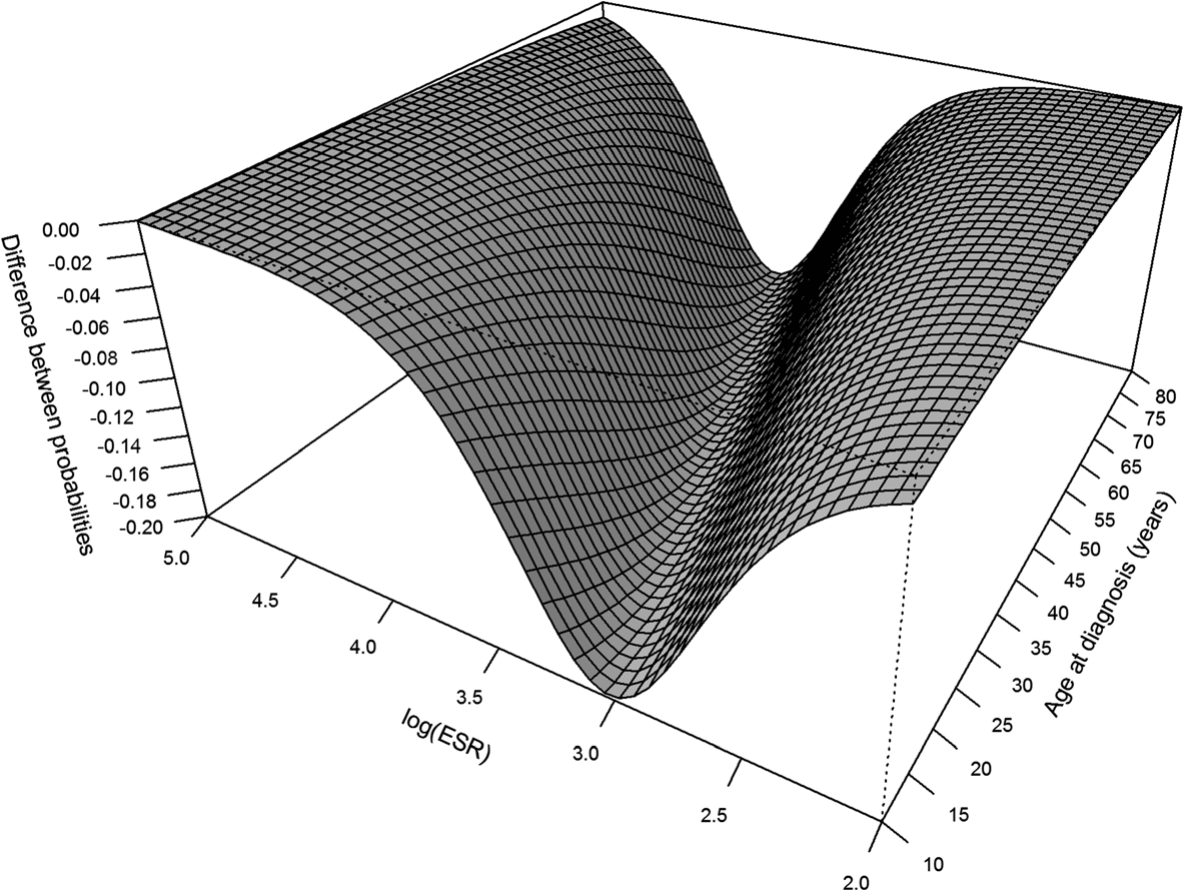
**Probability difference in MTX response between *KIR2DS4f *-positive and** ***KIR2DS4f*-negative patients with similar RA baseline.**

The effect of the *KIR2DS4f* gene on the probability of a response to MTX in RF- and ED-positive patients was strongest in individuals with medium values of the erythrocyte sedimentation rate (ESR), that is, log(ESR) about 3.0-3.5, i.e. 20-33 mm/h (Figure 2). Patients with high ESR values had a low probability of responding, and the presence or absence of *KIR2DS4f* did not affect their outcome, particularly in individuals with low age at onset. Patients with low ESR had a high probability of responding, which was not affected by the presence of *KIR2DS4f*, particularly in people with high age at onset (Figure 2). The *KIR2DS4f* effect did not depend on the presence of C1 (p = 0.209) or C2 (p = 0.484). Surprisingly, the age at diagnosis had a negligible influence on the *KIR2DS4f* effect.

## Discussion

This report confirms that almost one third of patients affected by rheumatoid arthritis do not respond to low-dose MTX treatment [15-18]. This phenomenon causes that the discovery of (bio)markers of response to the drug is still required for clinical practice. Here, we found a negative association of the *KIR2DS4f* gene with the response of RA patients to MTX treatment. This effect was visible not only in RF-positive, ED-positive patients who already had a lower chance of responding than negative ones, but also in RF- and ED-negative individuals who otherwise were better responders. Therefore, it is likely that the *KIR2DS4f* gene is expressed on effectors or positive regulators or inducers of autoimmune response, relatively resistant to the MTX effect. In this context, the CD4+CD28- subpopulation of T lymphocytes should be discussed. This T cell subset is rare in healthy individuals (about 1% of peripheral lymphocytes), but its expansion was observed in patients affected by, e.g., coronary artery disease [26], polycystic ovary syndrome [27], multiple sclerosis [28] and rheumatoid arthritis [4,29,30]. In contrast to those isolated from healthy volunteers [31], CD4+CD28- T cells from rheumatoid arthritis patients may exhibit autoreactive properties [4]. Instead of the CD28 molecule, these T cells frequently express activating KIR receptors, as was shown by Yen *et al.*[32] particularly for KIR2DS2 in RA vasculitis. Interestingly, it was suggested that the *KIR2DS4* gene may also be expressed on CD4+CD28- T lymphocytes [33], and could transmit an activating signal to effector cells. Therefore, *KIR2DS4f* gene-positive patients might have a potential to produce CD4+CD28-KIR2DS4+ T cells contributing to more severe forms of RA, which would be more resistant to therapy, including MTX treatment.

The gene mentioned above encodes the KIR2DS4f polyspecific receptor recognizing both HLA-A*11 and some HLA-C allotypes bearing C1 or C2 epitopes [10]. In Caucasians, including Poles, its gene is present in roughly one third of *KIR2DS4*-positive individuals, the other two thirds bearing its defective alleles, *KIR2DS4d* (see http://www.allelefrequencies.net database) [34], potentially encoding a soluble molecule which does not exhibit any activating properties [35]. However, in Far East Orientals, Japanese and Chinese, the proportions between *KIR2DS4f* and *KIR2DS4d* are reversed [33]. It would be interesting, then, to perform a similar analysis of RA patients’ response to MTX therapy in those populations, especially as the *KIR2DS4* gene seems to impact on RA susceptibility in Taiwanese patients [36].

Some studies suggest that ESR level, mentioned as a biomarker for chronic systemic inflammation as well as a parameter of disease activity, correlated with responsiveness to MTX (reviewed by Romao *et al.*) [37]. In the present study, we see that the effect of *KIR2DS4f* is strongest in individuals with medium susceptibility to the therapeutic effect of MTX characterized by an intermediate level of ESR. Patients with high ESR are resistant to therapy, and lack of the *KIR2DS4f* gene cannot help them; those with low ESR respond so well that even the presence of the *KIR2DS4f* gene does not cause any harm.

## Conclusion

This study shows that the presence of the full-length *KIR2DS4* gene reduces the probability that patients affected by rheumatoid arthritis will respond to methotrexate therapy. This phenomenon was strongest in individuals with a medium level (20-33 mm/h) of the erythrocyte sedimentation rate. The patient cohort with lower values of ERS responded better to MTX, while patients with a higher ESR level responded worse to therapy, and the presence of *KIR2DS4f* did not affect their outcome. Therefore, our findings, if confirmed using an independent and larger group of patients, might suggest that *KIR* typing could help to predict the response to MTX therapy, especially for patients with moderate rheumatoid arthritis.

## Abbreviations

CCP: Cyclic citrullinated peptide; ED: Erosive disease; ESR: Erythrocyte sedimentation rate; HLA: Human leukocyte antigen; KIR: Killer cell immunoglobulin-like receptor; LRS: Likelihood ratio statistic; MTX: Methotrexate; NK: Natural killer; OR: Odds ratio; RA: Rheumatoid arthritis; RF: Rheumatoid factor.

## Competing interests

The authors declare that they have no competing interests.

## Authors’ contributions

EM conducted the study and participated in the study design, data analysis and interpretation and drafting of the manuscript; AP participated in the data collection and drafting of the manuscript; DG contributed to the samples genotyping; PK participated in the study design, data analysis and interpretation as well as in the drafting of the manuscript. All authors read and approved the final manuscript.

## Pre-publication history

The pre-publication history for this paper can be accessed here:

http://www.biomedcentral.com/1471-2474/15/256/prepub
